# Risk Perception of HIV/AIDS and Low Self-Control Trait: Explaining Preventative Behaviors Among Iranian University Students

**DOI:** 10.5539/gjhs.v8n4p44

**Published:** 2015-07-30

**Authors:** Safooreh Esmaeilzadeh, Hamid Allahverdipour, Behrouz Fathi, Shayesteh Shirzadi

**Affiliations:** 1 Department of Health Education & Promotion, Tabriz University of Medical Sciences, Tabriz, Iran; 2 Clinical Psychiatry Research Center, Tabriz University of Medical Sciences, Tabriz 14711, Iran

**Keywords:** HIV/AIDS, self-control, Risk Perception, Extended Parallel Process Model

## Abstract

**Background:**

In spite of developed countries there are progressive trend about HIV/AIDS and its’ aspects of transmission in the low socio-economic societies. The aim of this was to explain the youth's behavior in adopting HIV/AIDS related preventive behaviors in a sample of Iranian university students by emphasizing on fear appeals approaches alongside examining the role of self-control trait for explaining adoption on danger or fear control processes based on Extended Parallel Process Model (EPPM).

**Methods:**

A sample of 156 randomly selected university students in Jolfa, Iran was recruited in a predictive cross-sectional study by application of a researcher-designed questionnaire through self-report data collection manner. Sexual high risk behaviors, the EPPM variables, self-control trait, and general self-efficacy were measured as theoretical framework.

**Results:**

Findings indicated that 31.3% of participants were in the fear control process versus 68.7% in danger control about HIV/AIDS and also the presence of multi-sex partners and amphetamine consumption amongst the participants. Low self-control trait and low perceived susceptibility significantly were related to having a history of multi-sex partners while high level of self-efficacy significantly increased the probability of condom use.

**Conclusion:**

Findings of the study were indicative of the protective role of high level of self-control, perceived susceptibility and self-efficacy factors on youth's high-risk behaviors and their preventative skills as well.

## 1. Introduction

HIV/AIDS as the most lethal infectious disease and the fourth mortality cause in the world with high enormous care costs, yet problematic for social life and targeting the young people, is of utmost intervention priorities of health systems (Etemad et al., 2007; Haghdoost et al., 2011). World Health Organization (2012) reported that 35.5 million people who suffering HIV/AIDS were living globally out of which 2.3 million cases were newly affected people (Haghdoost et al., 2011) and in 2012 about 1.6 million deaths have been occurred due to the AIDS (World Health Organization, 2013). Currently in most countries more than 39% of new cases of infections are seen among 15 to 24 years old youths (World Health Organization., 2013). In addition, according to the Ministry of Health report in Iran country the HIV/AIDS epidemic is expanding in the recent years (Ministry of Health and Medical Education [MOHME], 2014). The most recent evidences in Iran indicate that the number of people affected by HIV/AIDS has been reported to be about 27041 cases from which 89.3% were males and the most affected age groups were reported as being the 25-34 years old youths (45.9%) and also the most prevalent transmission ways included drug injection (68.1%) and sexually transmitted infection (12.7%) (MOHME, 2014).

In Islamic countries it seems like other countries youth people are more susceptible to high-risk behaviors like drug abuse, drunk driving, and high risk sexual contacts, and considering that due to the culture and normative behavioral patterns in the Islamic countries, which prohibits drinking alcohol and sexual contacts, the present evidences in some Islamic countries are indicative of the presence and increase in the high-risk sexual behaviors among youths (Rahmati et al., 2009). Kagimu et al (2012) considering the faith-based approach in prevention of HIV/AIDS among the 15-24 years old youths reported avoiding from sexual contacts among the Christian adolescents as equal to 54% and among the Muslim youth as 58% (Kagimu et al., 2012). In addition, Ajuwon et al. (2006) reported that among high school students in predominantly Muslim areas, 13% had experienced sexual intercourse out of which only (24%) had used a condom in their last sexual experience, which is indicative of their lack of low risk perception concerning HIV/AIDS disease, which can increase the spread of HIV/AIDS rate in such communities (Ajuwon et al., 2006).

Concerning HIV/AIDS risk perception a study on 20-24 years old youths showed that 32% of the girls and 38% of the boys do not consider themselves as vulnerable to the AIDS disease (Prata et al., 2006). Masvanya et al. (2009) in a study in Tanzania showed that although 54% of the students were sexually active, only 25% felt themselves to be at risk for AIDS (Maswanya et al., 2009). In addition, a study undertaken by Adefuye et al showed that the perceived risk of HIV infection in youths with high risk behaviors was in the low level (Adefuye et al., 2009). This is while the youth despite being aware of the dangers of risky behaviors, do not change their behavior toward healthy sexual behavior (Rahmati et al., 2009). The results of the studies suggest that willingness for dealing with the risk is multidimensional and its specific aspects include: Perceived threat and perceived efficacy. In fact, people need to be motivated, strengthen and gain support for changing high risk behaviors to healthy behaviors (Witte, 1992). One of the important manners to protect adolescents against HIV/AIDS is designing and developing effective fear appeal messages which a fear appeal messages effort to provoke the feeling of fear by depicting a personally relevant and significant threat—e.g. having an unsafe sex would be infected me by HIV virus and outlines feasible and effective recommendations to deter that threat—e.g. using condom will lead to a delightful life (Witte, 1998). In addition fear appeal messages would be useful if they contain an efficacy component as well as feeling of effectiveness of recommendations according to a theoretical frame (Allahverdipour et al., 2007; Glanz, Lewis, & Rimer, 2005; Witte, 1992a) and the Extended Parallel Process Model (EPPM) (Witte, 1992a) was emerged based on fear appeals theories. According to the EPPM, fear appeals initiate two appraisals. The first is the threat itself and the second assess the efficiency of suggested response (Witte, 1998; Witte, Meyer, & Martell, 2001). EPPM has been assessed useful for understanding adaptive behavior in the face of risk and according to this model, when people are faced with a health threat; they primarily assess the severity and perceived susceptibility of the threat (McMahan, Witte, & Meyer, 1998). If individuals do not consider themselves susceptible to risk or threat or assess themselves as invulnerable they will likely show no reaction to threat or risk but if after the threat assessment, they consider themselves as susceptible to that health risk or threat, they will likely deem the recommended solution as effective manner; and in this case, the message should be accepted by the individual and they will become motivated to danger control and if they will likely deem recommended solution as difficult, costly and time consuming and unenforceable, or controlling and managing them fear instead of trying to deal with the risk. In other words they will become motivated to control her/his fear (Witte, 1992; Witte & Allen, 2000).

In other side, analysis of the behavior of young people with a history of high risk behavior suggests that some of them have little control over their behavior. They tend to adopt high risk behaviors and compatibility with the high risk behaviors due to low self-control and without considering of the consequences (Lu, Yu, Ren, & Marshall, 2013; Vera & Moon, 2013). According to Gottfredson&Hirschi's theory, people with low self-control are susceptible to adopting high risk or imprudent behaviors like drug abuse, excessive alcohol consumption and high speed driving (Baron, 2003; Ellwanger, & Pratt, 2014; Ford & Blumenstein, 2012). Allahverdipour et al have reported that low self-control students have attempted drug abuse and cigarettes smoking under the influence of peer pressure (Allahverdipour et al., 2006). It has been reported in several other studies that low self-control among youth is a strong predictor of heavy alcohol consumption, smoking, drug abuse, higher levels of adventures, and risky behaviors (Adalbjarnardottir et al., 2002; Griffin et al., 2000; Wolfe, & Higgins, 2008). Additionally, it was found significant relationship between low self-control and alcohol consumption and the relevant adverse consequences (Robert et al., 2011). In addition, it was found more unprotected sexual behavior and engagement in sexual activity among people with low self-control (Hope & Chapple, 2004; Wills et al., 2003).

As it was not found study about relationship between self-control trait and risk perception with adopting HIV/AIDS preventative behaviors in Iran and because of increasing the rate of high risk sexual behaviors among Iranian youths, resulting in rapid growth in sexual transference of HIV/AIDS infection (MOHME, 2014), the present study has been undertaken aiming at analyzing the youth's behavior in adopting HIV/AIDS preventive behaviors through using a fear based model (EPPM) and examining the role of self-control in the adoption of risk control process or fear control.

## 2. Method and Materials

### 2.1 Participants and Procedure

This predictive cross-sectional study design was conducted in 2013 on 156 randomly selected non-medical students from among two universities of Jolfa and Hadishahr in north-west of Iran in the province of Azeribaijan. The inclusion criteria were all the under 28 years’ old students who registered for full term courses and had not history of mental disorders. The ethical approval of the study was granted by the Ethics Committee of Tabriz University of Medical Sciences and the participants’ informed consent was obtained. Data were collected by using self-reported questionnaires and 217 students were given a questionnaire out of which 61 questionnaires were discarded due to incomplete responses, finally 156 completed questionnaires were analyzed. In this study, the mean± SD of 22.15 ± 2.07 years with 64.7% male participation.

### 2.2 Measure

#### 2.2.1 Demographics

Demographic information included age, sex, marital status, field of study (management, computer, accounting, civil engineering) and lodging status (family, single home, dormitories).

#### 2.2.2 High Risk Behaviors

History of high risk behaviors included having multi-sex partners, having unsafe sex, refusal of proposed sexual behavior, tattoos, smoking, alcohol consumption, drug use, and company of friends who were addicted to drug use. Responses to all items were yes/no.

#### 2.2.3 EPPM Theoretical Variables

Witte's EPPM scale (Shiferaw, 2004; Witte et al., 2001) was modified for HIV/AIDS and 19 items were composed under four major constructs, (1) severity of HIV/AIDS consequences; (2) susceptibility to get HIV/AIDS; (3) self-efficacy and (4) response efficacy or evaluation of effectiveness of preventative behaviors. Four items were designed to measure perceived severity of HIV/AIDS consequences (e.g. “If I get AIDS, all my life will be ruined”). Two items were designed to measure perceived susceptibility to get HIV/AIDS (e.g. “I think I will not get AIDS during the next year”). Five items were designed to assess self-efficacy (e.g. “I believe that I am able to exercise restraints to prevent AIDS”). Five items were designed to evaluate response efficacy response efficacy or evaluation of effectiveness of preventative behaviors (e.g. “Using condoms can prevent the spread of AIDS”). Each of these items was measured on an ordinal 7-point Likert-type scale with 1=strongly disagree, to 7=strongly agree (at the opposite end of the continuum), and 4= neutral (in the middle). Estimated reliability coefficients for each EPPM variables were as follows: perceived susceptibility (α = 0.84); perceived severity (α =0.75); perceived response efficacy (α = 0.724); and perceived self-efficacy (α = 0.81)], indicating internal consistency.

#### 2.2.4 Self-Control

Self-control was measured by a modified set of 5 items scale based on Allahverdipour et al. (2006) self-control scale. Example of items was” Before I do anything, I think and decide about what I want to do” which were coded from 1 to 7 (strongly disagree to strongly agree). Reliability coefficients were calculated for the self-control scale (α = 0.62) and demonstrated internal consistency.

### 2.3 Statistical Analysis

In statistical analysis, the Chi-square test was used for analysis in the case of categorical variables and the Group *t*-test was used for quantitative variables, once the normality of their distribution had been tested. To identify how EPPM and Self-control predict HIV/AIDS related behaviors, Logistic regression analysis models were fitted to the data (*P*<0.05). All statistical analyses were performed using version 16.0 of the statistical software package SPSS (SPSS Inc., Chicago, Illinois, USA).

## 3. Results

Evaluation of high-risk behaviors showed that 7.7% (12/156) of the participants had multi-sex partners. In addition, 28.8% (45/156) of the participants had history of smoking, 5.8% (9/156) had been done tattoo, 16.7% (26/156) of them reported history of alcohol consumption and %5.8 (9/156) psychoactive drug use, and also 11% (17/156) reported history of companionship with friends during drug abuse. Furthermore 32.6% (51/156) reported having unsafe-sex and 25.6% (40/156) confirmed that they had got propositions to have sex out of which 12.5% (5/40) resulted in having sex. Evaluation of the participants’ beliefs indicated that they have reported the following measures as effective ways of protection against HIV/AIDS infection by refusing unsafe sexual until marriage (89.4%), using condoms and avoiding from high risk behaviors (90.1%), and following the protective instructions in high-risk conditions (96%).

Analysis of the results based on the EPPM variables were indicative of the low mean score of the perceived susceptibility 5.94(SD=3.33) which is indicative of lack of risk perception to be infected by HIV virus in the future. However the mean score of perceived severity of 21.15 (SD=7.96) shows that the students strong belief about serious consequences of HIV/AIDS. Additionally, the mean score of perceived response efficacy and mean score of perceived self-efficacy were 28.65 (SD=5.07) and 27 (SD=5.54) respectively. The high score of perceived response efficacy can be indicative of strong belief in students as to effectiveness of primary prevention strategies. Moreover, analysis of the results in relation to the critical points showed that about 31.3% of the students were in the stage of fear control process and 68.7% of them in the stage of danger control process. EPPM suggests two paths when people get frightened due to receiving fear appeal message including a) motivating to control the danger inherent in the threat and b) motivating to control fears about the threat. In case of motivating to control the danger, they consider ways to eliminate or decrease the threat. Alternatively, when people are motivated to control their fear, they do not think more about danger and instead they focus on how to control their fear by denying or refusing the risk (Witte & Allen, 2000; Witte, Meyer, & Martell, 2001).

Additionally, high self-efficacy was a strong predictor of condom use in the high risk sexual relationships (P-value =0.03) which indicates that high self-efficacy odds of using condom in sexual relationships increased by 11% (Table 1).

**Table 1:** Unadjusted and adjusted regression analysis for self-control, perceived response efficacy, perceived self-Efficacy as predictors of condom use

Variables	Adjusted				Unadjusted			
	
	OR	95%CI		Sig	OR	95%CI		Sig
		LL	UL			LL	UL	
Self-control	1.01	0.93	1.10	0.71	1.01	0.92	1.11	0.76
Response Efficacy	1.05	0.96	1.15	0.22	1.00	0.90	1.11	0.90
Perceived Self-Efficacy	1.11	1.01	1.21	0.02	1.11	1.00	1.22	0.03

*Note.* OR= Odds Ratio; CI=confidence interval; LL=lower limit, UL=upper limit.

Regarding the self-control status, 17.8% of the males and 16.4% of the females were low self-control. In addition, 22.5% of the respondents with low self-control and 4.7% with high self-control stated that they had multi-sex partners (X^2^= 9.708, p-value = 0.002). Evaluation of the relationship between self-control status and attending with friends during drug abuse showed that 23.1% of the low self-control students had previous history of accompanying drug addicted friends versus 8.6% among the high self-control students (X^2^= 4.616, p-value =.032). Additionally, the previous history of alcohol consumption among students with low and high self-control was 33.3% and 13.2% respectively (X^2^= 6.530, p-value = 0.011).

Table 2 indicates results of the simple logistic regression (univariate) analysis for self-control (P-value = 0.04) and the perceived susceptibility which are strong predictors of multi-sex partners (P-value = 0.01). The findings showed that high self-control trait decreased chances of having multi-sex partners by 13% while the low perceived susceptibility of getting HIV/AIDS infection increased the chances of having multi-sex partners by 25%. In the next stage, all constructs were entered into the multivariate logistic regression test and data analysis showed that, the high self-control trait significantly decreased risk of having multi-sex partners by 19% while high perceived susceptibility of getting HIV/AIDS increased it by 34%.

**Table 2:** Unadjusted and Adjusted Regression Analysis for Self-Control, Perceived Response, Efficacy Perceived Self-Efficacy, Perceived Severity of HIV/AIDS consequences, and Perceived Susceptibility to get HIV/AISD as Predictors of Multi-sex partners

Variables	Adjusted				Unadjusted			
	
	OR	95%CI		Sig	OR	95%CI		Sig
		LL	UL			LL	UL	
Self-control	0.87	0.76	0.99	0.041	0.81	0.70	0.94	0.008
Response Efficacy of preventive manners	1.17	0.99	1.37	0.06	1.20	0.97	1.49	0.081
Self-Efficacy	1.08	0.96	1.22	0.18	1.05	0.90	1.22	0.47
Severity of HIV/AIDS	0.99	0.91	1.07	0.91	0.97	0.91	1.03	0.31
Susceptibility to get HIV/AIDS infection	1.25	1.04	1.50	0.014	1.34	1.06	1.7	0.01

*Note.* OR= Odds Ratio; CI=confidence interval; LL=lower limit, UL=upper limit.

In addition, as shown in Table 3, high level of self-control was a strong predictor of alcohol consumption history (sig: 0.009) and high level of self-control decreased the probability of alcohol consumption by 12%. In the next stage, all the significant and insignificant constructs of the initial stage were entered into the multivariate logistic regression test in which the low self-control trait significantly increased the probability of using condom in sexual relations by 15%.

**Table 3:** Summary of Unadjusted regression analysis and Odds Ratiofor self-control and EPPM theoretical variables as predictors of alcohol use

	Adjusted				Unadjusted			
	
	OR	95%CI		Sig	OR	95%CI		Sig
		LL	UL			LL	UL	
Self-Control	0.88	0.80	0.96	0.009	0.85	0.77	0.94	0.003
Perceived Response Efficacy	1.08	0.98	1.20	0.092	1.12	0.98	1.27	0.084
Perceived Self-Efficacy	1.02	0.94	1.11	0.52	0.99	0.90	1.10	0.96
Perceived Severity	1.00	0.95	1.05	0.85	0.98	0.93	1.03	0.50
Perceived Susceptibility to get HIV/AIDS	1.10	0.97	1.25	0.11	1.10	0.95	1.27	0.18

*Note.* OR= Odds Ratio; CI=confidence interval; LL=lower limit, UL=upper limit.

## 4. Discussion

Findings of current study are confirmatory to the potential of the self-control, perceived susceptibility and self-efficacy to explain high-risk behaviors. In other words, the findings indicated how it is possible to put self-control into EPPM and interpret the reciprocal relation between EPPM theoretical variables and self-control status.

The present study indicated the presence of multi-sex partners, alcohol use, and amphetamine consumption among Iranian students is consistent to the studies undertaken in the same context in other Islamic countries (Ajuwon et al., 2005; Kagimu et al., 2012; Maswanya et al., 2009; Mohammad et al., 2007; Mohammadi et al., 2006; Raheel et al., 2013). Furthermore, self-efficacy was the only predictor of using condoms in high risk sexual relations from among the EPPM theoretical variables and added self-control construct. Bandura (1995) believes that there are numerous activities that if performed suitably, will result in favorable outcomes, but people who are skeptical of their abilities, are incapable of doing such acts. In fact, people with high self-efficacy expect achieving success in their efforts towards their goals and such people expect desirable consequences out of performing a special behavior (Bandura, 1995). Perceived self-efficacy conceptualized to consider about goals and the expected outcomes of an individual which could be effective in conducting, abandoning or prevention from certain high risk behaviors (Marlatt, Baer, & Quigley, 1997). It seems that if someone recognizes more benefits than disadvantages of using condoms in high risk sexual relationships, s/he will gain the ability to use condoms; otherwise, as Di Clemente et al have concluded, a person with low self-efficacy will involve in problematic behaviors and will remain engaged in one of the behavior change steps, in order that S/he would fail moving towards moderating the problematic behavior (DiClemente, Fairhurst, & Piotrowski, 1995).

As HIV/AIDS risk perception was the main object of the present study. Low level of perceived susceptibility to be infected by HIV virus indicated that the students feel low risk or probability of being infected by HIV virus in the future which is similar to the results of the study carried out by Ijadunolaet al. (2007) in Nigeria in which 85% of the students considered themselves at low if not zero risk of such infection while 77% were at actual risk of vulnerability to HIV/AIDS disease (Ijadunola et al., 2007). Additionally, results of the study in Ghana by Ganle et al. (2012) reported that despite a high level of awareness concerning HIV/AIDS among youth people, they have some misconceptions such as low risk perception of HIV infection, being optimistic about not being infected with HIV, and having history of frequent high risk sexual contact (Ganle et al., 2012). In this regard, Weinstein asserts that people often evaluate themselves at lower risks for negative events in life than their peers (Weinstein, 1980).

optimistic bias among youth is known as one of the important effective reasons that make youth willing to engage in high risk behaviors (Maxwell, 1996). Optimistic bias or sense of invulnerability is defined as a judgment that the individual feel himself/herself to experience a definite bad or dangerous situation or their adverse consequences lower than the others (Klein, & Helweg-Larsen, 2002; Price, Pentecost, & Voth, 2002). Sohn et al. (2012) reported optimistic bias among the Korean youths concerning vulnerability to HIV/AIDS and also they reported relationship between self-esteem and self-control and their predictor role in strengthening the optimistic bias (Sohn et al., 2012). Additionally, Klein et al reported the significant relationship between perceived control and optimistic bias (Klein, & Helweg-Larsen, 2002).

Additionally, there are difference between Self-control which it is known as the ability to control one's emotions, behavior, and desires in the face of external demands in order to function in society (Gottfredson & Hirschi, 1990) and self-efficacy which it is defined as the extent or strength of one's belief in one's own ability to complete tasks and reach the desired goals (DiClemente, Fairhurst, & Piotrowski, 1995). In other words, low self control is related to deviant behaviors while low self-efficacy is related to inability to complete the requested or planned performance. Low self-control trait and its consequences are the other problems that lead to high risk behaviors among youth people. Based on the findings of the present study, there was a significant relationship between low self-control and multi-sex partners as well as company with the friends with previous history of marijuana smoking, meanwhile the self-control was a predictor of multi-sex partners among the students which indicates that numerous studies are consistent with findings of current study in emphasizing that high-risk behaviors occur more frequently among people with low self-control (Adalbjarnardottir & Rafnsson, 2002; Allahverdipour et al., 2006; Lu et al., 2013; Sussman, Dent, & Leu, 2001; Vera, & Moon, 2013).

Gottfredson and Hirschi’ sgeneral theory of crime is based on the premise that individuals with low self-control were more likely to involve in risky behaviors such as drug abuse, smoking, and unprotected sexual behaviors (Ford & Blumenstein, 2012; Hope & Chapple, 2004; Wiederman, 2004). Studies on self-control theory have shown that low self-control trait lead to direct and indirect effects on the alcohol consumption as well as drug abuse (Klein & Helweg-Larsen, 2002; Wiederman, 2004). Additionally, Allahverdipour et al. reported a significant inverse relationship between low self-control trait and behavioral intention toward drug abuse among the Iranian high school students (Allahverdipour et al., 2006) and also Ford et al. (2013) showed relationship between low self-control and several forms of substance use including binge drinking, marijuana use, and prescription drug misuse and other illicit drug use. In addition self-control was known as a predictor of unprotected sexual behavior among young male and involvement in the unprotected sexual relationship (Hope & Chapple, 2004; Wills et al., 2003).

This study had a number of limitations. First, the study used self-reported measures which may be subject to inaccurate recall and/or reporting. Second, the results cannot be generalized to all Iranian students because it was carried out only in Northwest of, Iran. Additionally, low reliability coefficient of Self-control scale was another limitation of this study, which may affects on accuracy of results related to self-control.

## 5. Conclusion

The findings emerged from current study consistent with previous evidences signify the progressive trend of high risk behaviors among youth people specially increasing unsafe-sex in conjunction with high prevalence rate of HIV/AIDS, indicating necessity for the containment of which the interventions for promoting their ability to prevention must be put on high priority of health program agenda and health services as well. Additionally, the emphasis on the self-control factor in health education programs designed for the youth could play a decisive role in effectiveness of the health promoting programs where our audiences are youth people.
